# Dense KNN Polycrystals Doped by Er_2_O_3_ Obtained by Hot Pressing with Hexagonal Boron Nitride Protective Layer

**DOI:** 10.3390/ma13245741

**Published:** 2020-12-16

**Authors:** Paweł Rutkowski, Jan Huebner, Adrian Graboś, Dariusz Kata, Bogdan Sapiński, Marek Faryna

**Affiliations:** 1 Department of Ceramics and Refractories, Faculty of Materials Science and Ceramics, AGH University of Science and Technology in Krakow, al. Mickiewicza 30, 30-059 Kraków, Poland; pawel.rutkowski@agh.edu.pl (P.R.); agrabos@agh.edu.pl (A.G.); kata@agh.edu.pl (D.K.); 2 Department of Process Control, Faculty of Mechanical Engineering and Robotics, AGH University of Science and Technology, al. Mickiewicza 30, 30-059 Kraków, Poland; deep@agh.edu.pl; 3 Institute of Metallurgy and Materials Science, Polish Academy of Sciences, ul. Reymonta 25, 30-059 Kraków, Poland; m.faryna@imim.pl

**Keywords:** KNN, niobate, piezoelectrics, h-BN, hot-pressing, Er_2_O_3_

## Abstract

Analysis of dense Potassium Sodium Niobate (KNN) ceramic obtained by hot pressing (HP) method at 1100 °C are presented in this paper. The synthesis of KNN-based piezoelectrics meets the following challenges—low density of material, uncontrolled K/Na ratio, multiphase composition and formation of different KNN structures. The classical hot pressing approach results in contamination by carbon originating from graphite molds. The proposed hexagonal Boron Carbide (h-BN) layer between green sample and graphite mold could protect samples from carbon contamination. Additionally, the presence of h-BN may decrease the formation of oxygen vacancies, which allows us to maintain the semiconductor features of the KNN structure. Remaining issues were addressed with the addition of excess Na and Er_2_O_3_ doping. The results showed that excess Na addition allowed us to compensate evaporation of sodium during the synthesis and sintering. Er_2_O_3_ was added as sintering aid to limit abnormal grain growth caused by h–BN addition. The modification of amount of Na and Er_2_O_3_ addition resulted in high purity KNN samples with tetragonal structure and apparent density higher than 97%. Finally, piezoelectric features of prepared dense samples were measured and presented.

## 1. Introduction

Ferroelectric materials are widely used as a piezoelectric ceramics, serving as sensors, motors, energy harvesters or parts of advance measurement devices among many other applications [1,2,3,4]. Their prominent piezo- and ferroelectric properties derived from the perovskite crystal structure are still utilized in new emerging fields of engineering, leading to new solutions and possibilities. However, those materials are also a current challenge for many researchers as their most commonly used element, lead, is proven to be dangerous to natural environment [5]. Therefore Pb-based piezoelectrics, such as PbZr_x_Ti_1−x_O_3_ (PZT), Pb_1−x_La_x_(Zr_1−y_Ti_y_)_1−x/4_O_3_ (PLZT) or Pb(Mg_1/3_Nb_2/3_)O_3_ although dominating at this moment, are aimed to be replaced with lead-free solutions.

Ferroelectric materials, such as described above (except for Polyvinylidene Fluoride-PVDF), used as piezoelectrics have the crystalline structure of perovskite, which is commonly known as a structure similar to BaTiO_3_. Its specific positioning combined with proper compositions stands for many functional properties, including ferromagnetism or thermoelectricity. KNN materials consist of two phases: ferroelectric potassium niobate (K_1−x_NbO_3_) and antiferroelectric sodium niobate (Na_x_NbO_3_) which differ in transition but crystallize in the almost the same perovskite structure. The basic transition dependency of K/Na ratio results in possibility of obtaining functional perovskite structure with outstanding piezoelectric properties, given that mentioned stoichiometric proportions are preserved, because of morphotropic phase boundary (MPB) that it creates in the tetragonal-rhombohedral transition [6].

The main challenge of implementing KNN lead-free piezoelectric materials is the proper synthesis. While material can be relatively simply obtained by high temperature processing of uniaxially pressed samples, its expected properties could be decreased due to the small density of samples. Amongst possible reasons of this situation, most possible are: evaporation of sodium, alkali vacancies created during sintering and lack of liquid phase in the microstructure of pure KNN [7,8,9].

In face of those problems, a proper solutions were pursued by many researching teams experimenting with both environment and composition. Shimizu et al. showed that pure alkali niobate ferroelectric and dielectric properties can be significantly improved using only low oxygen partial pressure processes [10]. Those studies were inspired by articles on KNN ceramics sintered under low oxygen partial pressure [11], which showed suppressing of vacancies formation in used process, both proving that reducing oxygen interaction with those materials is desired on every step of technological process. Studies concerning sintering in H_2_ and Ar atmospheres respectively showed, that similar improvements of properties can be obtained by changing the environment [12]. Moreover, similar studies shown that different atmospheres can affect dopants behavior [9].

Yang et al., while obtaining transparent lead-free ferroelectric ceramics, went one step further, employing sealed sintering process and comparing it to sintering in air. Although used KNN materials were differing due to the doping of Ca(Sc_0.5_Nb_0.5_)O_3_ (CSN), it was shown, that sealed ceramic samples were denser that their unsealed counterparts. The overall improvement of electrical properties was also noted [13]. Additionally, more advanced, alternative processes were involved including, for example, spark plasma sintering (SPS) and while reducing volatility of obtained samples and offering overall better performance, they are still more technologically demanding [14].

In addition to process changes, various dopants were reported to improve KNN synthesis processes and were considered as a possible solution to this problem. The simplest idea of doping with alkaline earth metals was proposed by Malič et al. but was generally lacking significant, if any, positive effect on densification of samples [15]. Previously mentioned articles on low oxygen partial pressure in KNN processes involved the addition of LiF, to further improve the densification by usage of Lithium [11] with similar solution proposed by Saito et al. but additionally involving alkaline earth metals, resulting in advanced systems, such as: (K_0.44_Na_0.52_Li_0.04_)(Nb_0.86_Ta_0.10_Sb_0.04_)O_3_ in which lithium subtracts either potassium or sodium atoms, while alkaline earth metals are in niobium positions [16] and resulting in additional improvements of piezoelectric charge coefficients (d_33_) with relatively high conversion ratios. Due to those findings, later iterations of solution were to follow [17,18], showing similar increase of the same coefficients. Important process distinction was provided in studies, in which KNN was firstly annealed and then milled with different doping oxides. Such process improved densities of sample with relatively small additions of dopants [19].

Another chance of improvement came with the involvement of rare earth metals oxides. Those materials were expected to improve samples, based on previous research, performed to improve PZT material systems. Those studies showed that Yb^3+^ substitution in perovskite structure could reduce coupling factor of materials but greatly improve its other piezoelectric properties [20]. In case of KNN materials, coupling factor is considered high and therefore such dopant was considered in our previous studies prior to this paper [21] alongside Er_2_O_3_ which was investigated by Zhao et al. and shown that it could result in creation of highly desired liquid phase during sintering [22].

The aim of this study was to obtain a dense KNN-based material, using industrially applicable process, using the listed findings and preliminary studies performed prior to this paper, which would be tested in stress sensor. To achieve this task, dopants of rare-earth elements, namely Er_2_O_3_, were employed. In comparison to previous research [21], processing was improved by application of h-BN layer on samples that were then sintered by hot pressing (HP). Presence of h-BN were expected to prevent reaction between powders and carbon mold. Additionally, boron could diffuse to sintered material which is beneficial for its densification and grain growth. Finally, polarization of samples was performed to reorder dipoles in materials for better piezoelectric response. Usage of hot pressing in h-BN form would expectedly improve the density of material in comparison to commonly used techniques, additionally texturing the microstructure of samples, which is desirable in case of piezoelectrics [23]. The texturization of material is promoted by one axis pressing which enhances directed grain growth during sintering. Moreover, the decrease of sintering temperature may reduce the overall alkali evaporation, allowing to manufacture stoichiometric 0.5 KNN material when combined with additional sodium in initial powder mixture.

## 2. Materials and Methods

To synthesize KNN (K_0.5_Na_0.5_NbO_3_), the following commercial reactants were used—Nb_2_O_5_ powder of CHANGSHA EASCHEM CO. Ltd. (Changsha, China) and K_2_CO_3_, Na_2_CO_3_ carbonate powders produced by Lach-Ner (Neratovice, Czech Republic). In the first step of KNN synthesis, the carbonate reactants were annealed at 150 °C for 24 h in order to remove residual water. Then K_2_CO_3_, Na_2_CO_3_ and Nb_2_O_5_ powders with 5 wt.% and 10 wt.% excess of sodium carbonate were mixed in ceramic mortar. The extra amount of Na_2_CO_3_ was added to compensate sodium evaporation process during synthesis. Thus prepared powders were dry homogenized in ball mill using 10.0 mm diameter silicon nitride milling media for 24 h. The reactants mixture were placed into alumina crucible with lid and heated up in air atmosphere to temperature of 950 °C, in which they were annealed for 2 h. The crushed and grinded KNN reaction bed was analyzed by X-ray diffraction method. The qualitative and quantitative X-ray Diffraction (XRD)/Rietveld phase composition analysis was made using PANalytical (model) diffractometer (Malvern, UK), equipped with Cu anode supported by X-pert HighScore software (Almelo, The Netherlands) for quantitative analysis of the samples.

**Figure 1:**
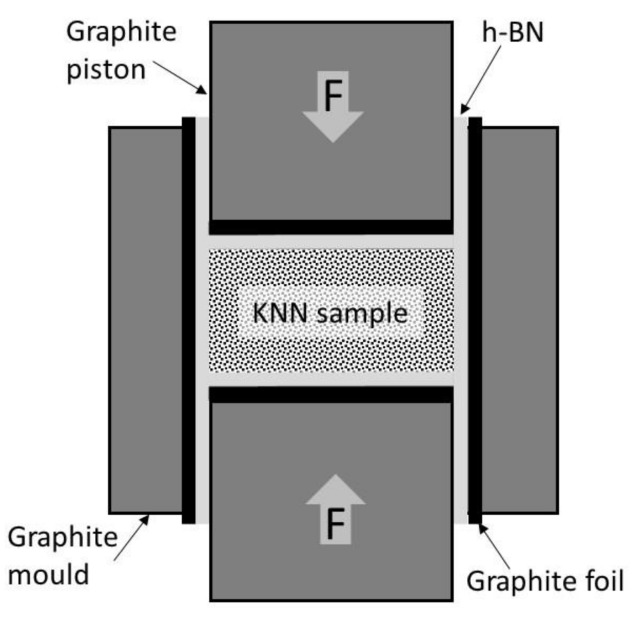
The hot-press graphite mold protected by graphite foil and sprayed h-BN layer.

The obtained KNN powders were then mixed with 1 wt.% and 2 wt.% of Er_2_O_3_ used as sintering activator produced by Ganzhou HongDe New Technology Development Ltd. (Ganzhou, China). having 0.65 m^2^/g specific surface area, 99.5% purity and 8.69 g/cm^3^ helium density. Due to company information average grain size of powder was estimated to d_50_ = 1.4 µm. The powder batches were dry homogenized in rotary mill using 5.0 mm Si_3_N_4_ grinding media for 24 h. Then mixtures were screened by 0.5 mm mesh sieve and placed in 25.0 mm diameter hot pressing graphite molds. To prevent excessive contamination of green samples by carbon, the graphite parts of the mold were covered by graphite foil and next it was sprayed by hexagonal boron nitride (h-BN) powder, as schematically showed in Figure 1. The additional positive effect of the h-BN usage is stimulation of KNN densification process [24].

Prepared powders were hot pressed (HP) under 25 MPa of pressure for 2 h at 1100 °C in argon flow with heating/cooling rate of 10 °C/min and 8 °C/min. respectively. The applied pressure accelerated sintering, polycrystal densification and reduced evaporation of sodium. The obtained dense KNN sinters had diameter of 25.0 mm and thickness of 5.0 mm. Additionally, polycrystalline KNN reference sample without sintering activator and Na_2_CO_3_ extra addition was manufactured in order to check: density, microstructure formation and phase composition. Cooling rate of HP process for those materials was very low of 3 °C/min. to reduce internal stresses caused by phase transformation and polycrystal formation during heat treatment.

The water contact with the sample is harmful due to the hygroscopic feature of KNN, for this reason, all samples handling was carefully proceeded in dry and no moisture conditions. The density measurements of KNN polycrystals was conducted in helium pycnometer AccuPyc II 1340 produced by Micrometrics Company (Norcross, USA). An average density value was calculated based on 30 measurement points. For microscopic observation purposes the sintered samples were included in resin and then polished by Struers polisher TegraPol-21 equipped with TegraForce-5 grinding discs under set load of 35 N and rotation speed of 300 rpm. The process was carried out in isopropanol environment. Surface preparation was done by diamond discs followed by final polishing with 0.25 um colloidal silica, which enabled mechanical etching of polycrystal to reveal microstructure details. Scanning electron microscope (SEM) FEI NOVA NANO SEM 200 (FEI, Acht, the Netherlands) was used for preliminary observations. Fracture morphology was recorded by use of secondary electrons (SE), while polished samples were observed in backscatter electron (BSE) mode. Detailed microstructural examinations were performed by use of scanning electron microscope FEI QUANTA 3D FEG SEM (FEI, Acht, the Netherlands) equipped with energy dispersive spectrometer (EDS) (EDAX, Tilburg, the Netherlands). The applied energy of the electron beam was 15 keV (sufficient enough to excite the maximum intensity of Er Lα line) and beam current of 11 nA. Microstructure observations and microbeam analysis was performed in low vacuum conditions. Carbon dioxide gas was used to eliminate any reaction of the KNN with water vapor, which is the default gas in Variable Pressure SEM measurements. ZAF correction procedure was used for calculations. The following standards have been used for quantitative analysis: NaCl crystal for Na Kα line, KBr for K Kα line, pure niobium for Nb Lα line, Al_2_O_3_ for O Kα line, Er_2_O_3_ for Er Mα and Er Lα lines. During calculations pressure variation method based on gas compensated technique was used. In order to confirm influence of HP applied pressure on material texturing, the reference sample was taken for ultrasonic measurement of longitudinal wave propagation in parallel and perpendicular directions to pressing axis. The ultrasonic examination was made by UZP-1 (INCO–VERITAS, Kraków, Poland) apparatus equipped with 1 MHz heads. In order to measure piezoelectric signal generated by samples in simulated real conditions, selected materials were subjected to poling process in custom built apparatus for 1 h in 2 kV/mm in silicon oil environment. It was followed by measurement of electric signal generated by piezoelectric properties of previously poled samples subjected to repetitive cyclic compression by custom built apparatus with force 150 N and 200 N for 2000 cycles. This equipment allows us to apply repetitive cyclic compression to rectangular or circular samples with set load between 100 to 1000 N. The analysis of these signals was performed by DasyLab 2016 software (measX GmbH, Moenchengladbach, Germany) in order to minimize distortions during measurements.

## 3. Result and Discussion for KNN Polycrystal without Sintering Aids

**Figure 2:**
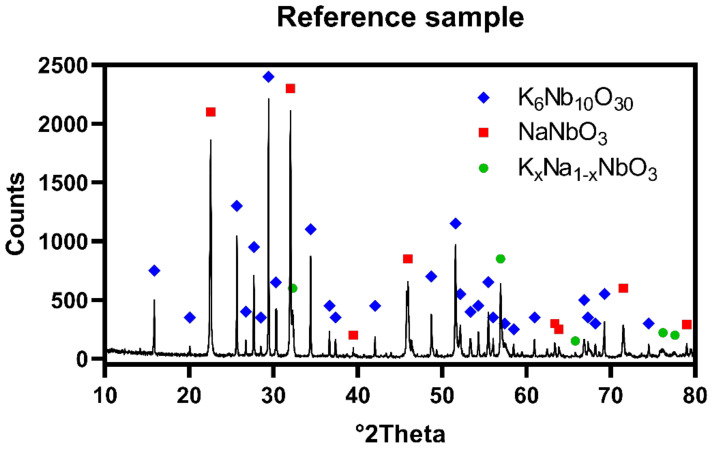
X-ray diffraction (XRD) pattern of reference sample hot-pressed without sintering additions, under 25 MPa of pressure for 2 h at 1100 °C in argon flow with heating/cooling rate of 10 °C/min and 8 °C/min, respectively.

The X-ray diffraction patterns obtained for reference sample revealed presence of two major phases: K_4_Nb_10_O_30_ in quantity of 83.5 wt.% and NaNbO_3_ in quantity of 11.5 wt.%, while planned stoichiometric K_x_Na_1−x_NbO_3_ existed as minor phase in quantity of approximately 5.0 wt.%. That may be caused by deficiency of sodium in the system, due to evaporation occurred at both synthesis and hot-pressing steps. Figure 2 shows XRD pattern of prepared polycrystal, respectively.

**Figure 3:**
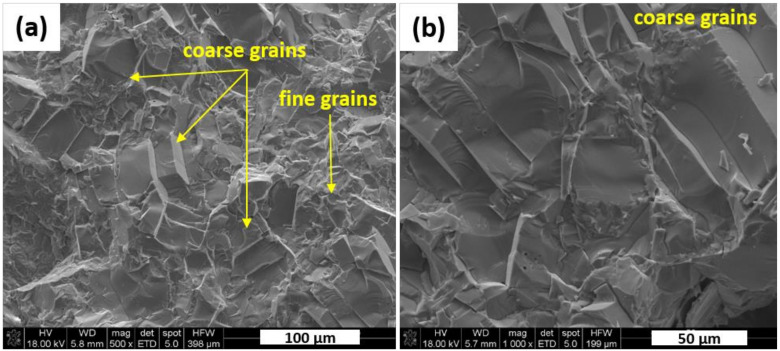
Fracture morphology of the reference sample: (**a**) bimodal grain size distribution, (**b**) microstructure details of coarse grains; SE mode in both cases.

Relative density of manufactured reference material was estimated as 98.4% based on the theoretical one of 4.55 g/cm^3^ measured by helium method on the polycrystalline grinded sample. Then, the material was examined by SEM. Morphological features of KNN fracture are presented in Figure 3. It shows the presence of coarser grains (approx. 50 µm) along with the smaller ones (approx. 5 µm) in close proximity to each other. Thus, SEM observations of hot pressed reference sinter presented in Figure 3 confirmed the existence of bi-modal grain size distribution. Those larger grains are textured in a perpendicular direction to the pressing axis. That suggests a favored formation of flattened grains under uniaxial load applied by hot pressing.

The ultrasonic analysis of longitudinal wave propagation examined in perpendicular directions of the reference sinter confirms texturing influence of HP process, what is visible in the wave propagation anisotropy reaching 25%. The measurement directions and wave velocity are collected in Table 1. This agrees with SEM observations that shown specific morphology of larger grains after hot pressing of powders.

## 4. Results and Discussion for Erbium Oxide Doped KNN

In order to obtain materials containing K_0.5_Na_0.5_NbO_3_, Er_2_O_3_ sintering activator was added because of its positive effect in formation of both orthorhombic and tetragonal structures which enhance piezoelectric properties of obtained sinters [21]. The hot pressed KNN samples containing 5 wt.% or 10 wt.% excess amount of Na_2_CO_3_ and addition of 1 wt.% or 2 wt.% of Er_2_O_3_ were subjected to density measurements with results collected in Table 2. The helium density was above 4.43 g/cm^3^, which confirmed that all of samples have more than 97% of relative density which is enough for piezoelectric generator manufacturing. This suggests that material may be strengthened because of low concentration of microstructure defects. Complete density data is presented in Table 3 and it indicates that higher amounts of Er and Na in the material might lead to a slight decrease in material density.

**Figure 4:**
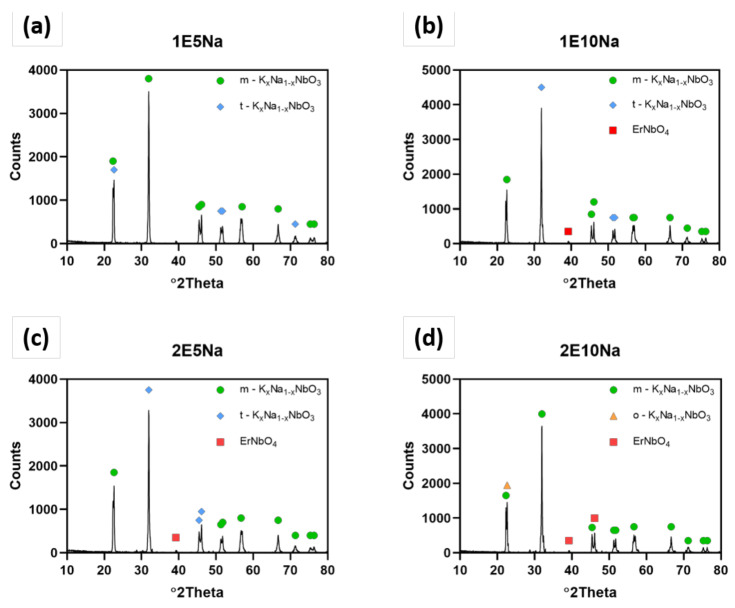
XRD patterns for all hot pressed samples with addition of Er2O3 and excess amount of Na2CO3 under 25 MPa of pressure for 2 h at 1100 °C in argon flow with heating/cooling rate of 10 °C/min and 8 °C/min, respectively; (**a**) 1 wt.% of Er_2_O_3_ and 5 wt.% of NaCO_3_ excess, (**b**) 1 wt.% of Er_2_O_3_ and 10 wt.% of NaCO_3_ excess, (**c**) 2 wt.% of Er_2_O_3_ and 5 wt.% of NaCO_3_ excess, (**d**) 2 wt.% of Er_2_O_3_ and 10 wt.% of NaCO_3_ excess

The results of quantitative and qualitative XRD analysis presented in Figure 4 and Table 3 showed that both: excess amount of Na_2_CO_3_ and Er_2_O_3_ sintering aid allowed us to obtain almost pure KNN material in the tetragonal crystallographic structure. The increase of Er_2_O_3_ from 1 wt.% to 2 wt.% content and excess of Na_2_CO_3_ addition from 5 wt.% to 10 wt.% led to orthorhombic structure of K_0.5_Na_0.5_NbO_3_ and small quantities of KNN doped by erbium. However, the same mixture with both additions of 10 wt.% of Na_2_CO_3_ and 2% Er2O_3_ resulted in shift to orthorhombic structure. Lattice parameters of detected samples are gathered in Table 4. All phases has similar lattice parameters for all obtained sinters, with no significant differences between them. It confirms that hot pressing has no effect on lattice parameters.

**Figure 5:**
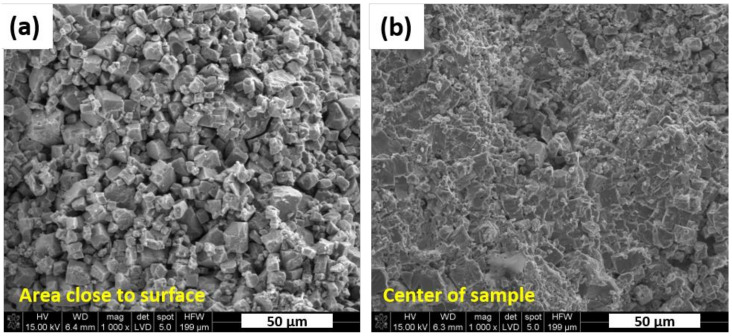
Fracture morphology recorded from two areas in the N5E1 sample: (**a**) the area close to surface of sample covered by h–BN layer during hot pressing (coarse grains), (**b**) the area in the center of the sample (fine grains); SE mode in low vacuum.

The obtained KNN sinters were subjected to microstructural and chemical analysis by SEM-EDS method. The material fracture with addition of 1 wt.% of Er_2_O_3_ is presented in Figure 5. It reveals the area close to surface that was covered by the h-BN protective layer during hot pressing, while Figure 5b shows the area in the center of the sample. Their morphological features are significantly different. It was observed that material in the center of sintered samples was characterized by much finer grains. It suggests that h-BN deposited on sample surface reacted with powders during sintering. As a result, abnormal grain growth, typical for KNN ceramics [25,26], was observed in close proximity to the surface. This phenomenon could be enhanced by the presence of boron, which has a positive effect both on densification and grain growth during thermal processing [24].

Results of average elemental composition measured by EDS technique for KNN taken from two randomly chosen flat areas of the polished N5E1 sample are collected in Table 5. They confirm a high amount of Nb in each of these sites. The Na to K atomic ratio was about 0.99 for the area 1 and about 1.98 for the area 2. Overabundance of Na caused by addition of 5 wt.% of Na_2_CO_3_ resulted in non-stoichiometric composition of KNN in some parts of the material. The nucleation rate of fine grains was previously confirmed to be enhanced by the excess amount of Na_2_CO_3_ introduced to the system [21].

**Figure 6:**
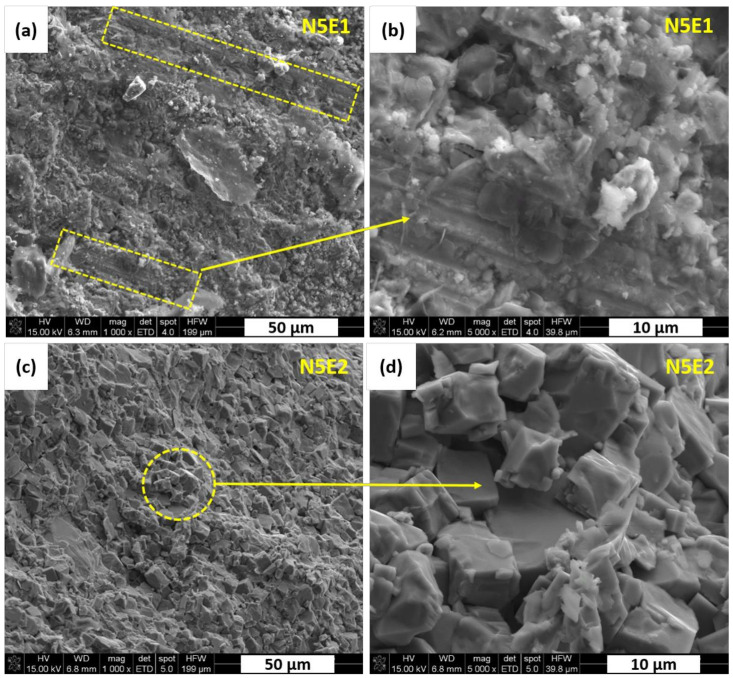
KNN polycrystals fractures: (**a**,**b**) N5E1 sample (**c**,**d**) N5E2 sample; all images recoded in SE mode.

The N5E1 polycrystal is characterized by some elongated and geometrically oriented grains as marked in Figure 6a,b. They are surrounded by fine grains with the size of about 1.0 µm. Higher content of Na_2_CO_3_ excess from 5 wt.% to 10 wt.% resulted in a much more uniform fractures morphology as shown in Figure 6c,d. Those grains have regular shapes with size of approx. 10 µm. No significant changes in microstructure details was observed by increased addition of Er_2_O_3_ from 1 wt.% to 2 wt.%. Based on the results of our previous work [21], it can be concluded that those fine grains of tetragonal K_x_Na_1−x_NbO_3_ were formed due to higher sodium content, which was enhanced by the excess amount of Na_2_CO_3_ introduced to the system.

**Figure 7:**
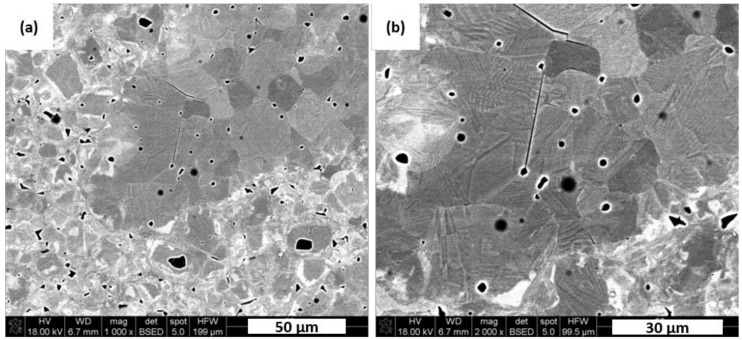
Microstructure domain of the N5E1 sample with 5% of Na_2_CO_3_ and 1% of Er_2_O_3_ addition revealed on polished and mechanically etched surface: (**a**) bimodal character of grain distribution, (**b**) details of spherical porosity caused by sodium evaporation; both images recorded in BSE mode.

Figure 7 presents SEM images of typical microstructure and domain patterns for a manufactured dense KNN polycrystals. Majority of visible grains have an average size of about 10–12 µm but there are also fine particles in size of less than 2 µm. Those coarse grains show simple parallel domain stripes that run through the whole grain in the etching plane. It is well known that the dielectric and piezoelectric properties of a ferroelectric ceramics could be resolved into intrinsic and extrinsic contributions. The intrinsic one is attributed to the relative ion shift that preserves the ferroelectric crystal structure, whereas the extrinsic one is related to the movement of domain walls [27]. Thus domain structure may have significantly positive influence on the piezoelectric and dielectric properties of a ferroelectric ceramics. It was expected that obtained dense polycrystals poses high piezoelectric feature. As evidence of sodium evaporation during processing or as a sintering result, some spherical pores remained in the microstructure as shown in Figure 7.

**Figure 8:**
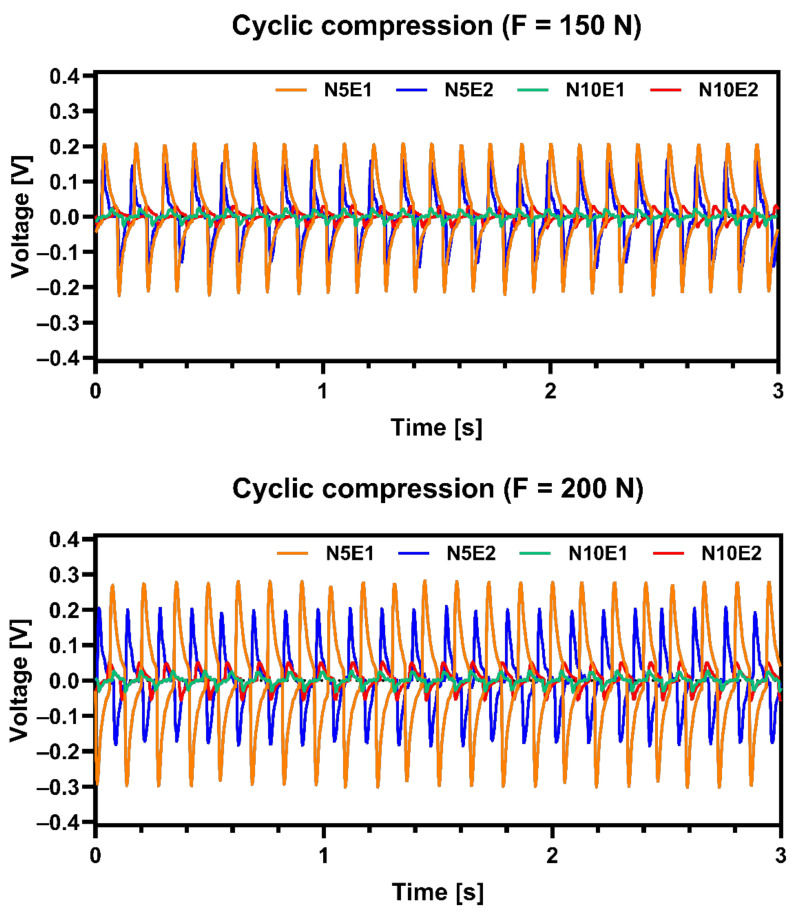
Signal generated by obtained samples under cyclic compression of 150 N (**above**) and 200 N (**below**).

Microstructure of sample N5E1 should have the best piezoelectric properties from all prepared samples thanks to its tetragonal crystallographic structure as shown in Table 4. Presented results were subjected to signal filtering in order to minimize effect of interference from electrical network. It was confirmed by measurements of electric signal generated by obtained samples shown in Figure 8. Results shows that N5E1 material generated highest voltage under compression for both 150 N and 200 N load. The second best was N10E1 which signals were slightly lower. Both samples with 10 wt.% excess of Na_2_CO_3_ exhibited much lower values. This proves that small overabundance of Na in materials can be beneficial for piezoelectric properties but must be carefully optimized.

## 5. Conclusions

The layer of h-BN on green samples was initially introduced as protective layer to prevent the excessive carbon contamination during hot pressing. Morphology of the samples in the areas close to sample surface showed a formation of much larger grains comparing to the grains in the central area of samples, which could result from limited diffusion of boron inside the samples during hot pressing. The excess amount of Na_2_CO_3_ added to powder mixtures have a positive influence on maintaining the desired tetragonal K_x_Na_1−x_NbO_3_ stoichiometry. Material with 5 wt.% excess of Na_2_CO_3_ and 1 wt.% of Er_2_O_3_ (N5E1 sample) which possess domain microstructure, generated highest electric signal of all samples for both 150 N and 200 N load cyclic compression. Combination of 10 wt.% Na_2_CO_3_ and 2 wt.% Er_2_O_3_ (N10E2 sample) additions led to extensive formation of monoclinic KNN with low piezoelectric properties. The hot pressing process applied in KNN manufacturing stimulated texturing of microstructure, which was confirmed by ultrasonic measurements indicating anisotropy of properties. In case of reference sample, an addition of hexagonal boron nitride leads to sintering intensification and extensive grain growth up to 50 µm. The experiment proved that it is possible to obtain KNN polycrystalline samples characterized by large dimensions. The diameter of obtained sinters was of 25 mm with thickness of 5 mm. The main phases detected were either orthorhombic or tetragonal KNN. Obtained sinters generated relatively high piezoelectric signal under cyclic compression.

## Figures and Tables

**Table 1:** Results of ultrasonic measurements for reference sample show texturing influence of hot pressing.

Measurement Direction	Wave Velocity (m/s)	Standard Deviation (SD) (m/s)	z/x Wave Velocity Relation	Young Modulus (GPa)	Standard Deviation (SD) (GPa)	z/x Young Modulus Relation
x	4243	12	1.33	80	0.1	1.78
z	5660	181		145	0.2	

**Table 2:** Density of hot pressed KNN with Er_2_O_3_ addition.

Sample ID	Sodium Carbonate Excess (wt.%)	Er_2__O__3_ Sintering Aid (wt.%)	Helium Density (g/cm^3^)	Standard Deviation (SD) (g/cm^3^)	Relative Density (%)
N5E1	5	1	4.4989	0.0069	98.8
N5E2	5	2	4.4492	0.0043	97.5
N10E1	10	1	4.4527	0.0082	97.8
N10E2	10	2	4.4327	0.0235	97.1

**Table 3:** Phase composition and crystallographic structure of obtained KNN polycrystals.

Sample ID	Na_2_CO_3_ Excess (wt.%)	Er_2_O_3_ Amount (wt.%)	Phase Composition (wt.%)			
			t-KNN	m-KNN	o-KNN	ErNbO_4_
N5E1	5	1	40.9	59.1	-	-
N5E2	5	2	58.0	40.5	-	1.5
N10E1	10	1	63.7	34.9	-	1.4
N10E2	10	2	76.9	-	20.2	2.9

**Table 4:** Lattice parameters of phases detected by XRD.

Sample ID	Phase	a	b	c
**N5E1**	m-KNN	5.62613	3.94295	5.657941
	t-KNN	3.95129	3.65129	3.99628
**N10E1**	m-KNN	5.63662	3.94715	5.66944
	t-KNN	3.94648	3.94648	4.00196
	ErNbO_4_	7.03888	10.90967	5.08305
**N5E2**	m-KNN	5.561942	3.94812	5.65664
	t-KNN	3.93872	3.93872	4.00164
	ErNbO_4_	7.02641	10.92769	5.06693
**N10E2**	m-KNN	5.63596	3.94296	5.66501
	o-KNN	5.54164	5.61758	15.61742
	ErNbO_4_	7.03112	10.91336	5.0688

**Table 5:** An average elemental composition measured by EDS for KNN taken from two flat areas of the polished N5E1 sample.

Area	Nb		Na		K		O		Er	
	(wt.%)	(at.%)	(wt.%)	(at.%)	(wt.%)	(at.%)	(wt.%)	(at.%)	(wt.%)	(at.%)
1	53.9 ± 1.1	20.3 ± 0.4	6.5 ± 0.3	9.9 ± 0.4	11.2 ± 0.4	10.0 ± 0.4	27.3 ± 0.5	59.6 ± 1.2	1.1 ± 0.5	0.2 ± 0.1
2	51.5 ± 1.0	17.6 ± 0.7	8.1 ± 0.3	11.1 ± 0.4	6.9 ± 0.3	5.6 ± 0.2	33.1 ± 0.7	65.6 ± 1.3	0.4 ± 0.2	0.1 ± 0.1
